# Developmental Self-Construction and -Configuration of Functional Neocortical Neuronal Networks

**DOI:** 10.1371/journal.pcbi.1003994

**Published:** 2014-12-04

**Authors:** Roman Bauer, Frédéric Zubler, Sabina Pfister, Andreas Hauri, Michael Pfeiffer, Dylan R. Muir, Rodney J. Douglas

**Affiliations:** 1 Institute of Neuroinformatics, University/ETH Zürich, Zürich, Switzerland; 2 School of Computing Science, Newcastle University, Newcastle upon Tyne, United Kingdom; 3 Department of Neurology, Inselspital Bern, Bern University Hospital, University of Bern, Bern, Switzerland; 4 Biozentrum, University of Basel, Basel, Switzerland; Indiana University, United States of America

## Abstract

The prenatal development of neural circuits must provide sufficient configuration to support at least a set of core postnatal behaviors. Although knowledge of various genetic and cellular aspects of development is accumulating rapidly, there is less systematic understanding of how these various processes play together in order to construct such functional networks. Here we make some steps toward such understanding by demonstrating through detailed simulations how a competitive co-operative (‘winner-take-all’, WTA) network architecture can arise by development from a single precursor cell. This precursor is granted a simplified gene regulatory network that directs cell mitosis, differentiation, migration, neurite outgrowth and synaptogenesis. Once initial axonal connection patterns are established, their synaptic weights undergo homeostatic unsupervised learning that is shaped by wave-like input patterns. We demonstrate how this autonomous genetically directed developmental sequence can give rise to self-calibrated WTA networks, and compare our simulation results with biological data.

## Author Summary

Models of learning in artificial neural networks generally assume that the neurons and approximate network are given, and then learning tunes the synaptic weights. By contrast, we address the question of how an entire functional neuronal network containing many differentiated neurons and connections can develop from only a single progenitor cell. We chose a winner-take-all network as the developmental target, because it is a computationally powerful circuit, and a candidate motif of neocortical networks. The key aspect of this challenge is that the developmental mechanisms must be locally autonomous as in Biology: They cannot depend on global knowledge or supervision. We have explored this developmental process by simulating in physical detail the fundamental biological behaviors, such as cell proliferation, neurite growth and synapse formation that give rise to the structural connectivity observed in the superficial layers of the neocortex. These differentiated, approximately connected neurons then adapt their synaptic weights homeostatically to obtain a uniform electrical signaling activity before going on to organize themselves according to the fundamental correlations embedded in a noisy wave-like input signal. In this way the precursor expands itself through development and unsupervised learning into winner-take-all functionality and orientation selectivity in a biologically plausible manner.

## Introduction

In this paper we address the question of how progenitor cells of the neocortical subplate can give rise to large functional neuronal sub-networks in the developed cortex. We choose winner-take-all (WTA) [1], [2] connectivity as the target of this self-construction and -configuration process because these sub-networks are consistent with the observed physiology [3], [4] and connectivity [5], [6] of neurons in the superficial layers of neocortex, and because they are powerful elements of computation [7], [8]. WTA networks actively select the strongest of multiple input signals, while suppressing the weaker ones. This fundamental characteristic is applicable in various contexts, and so many studies modeling cortical function are based on WTA modules [8]–[15].

**Figure 1:**
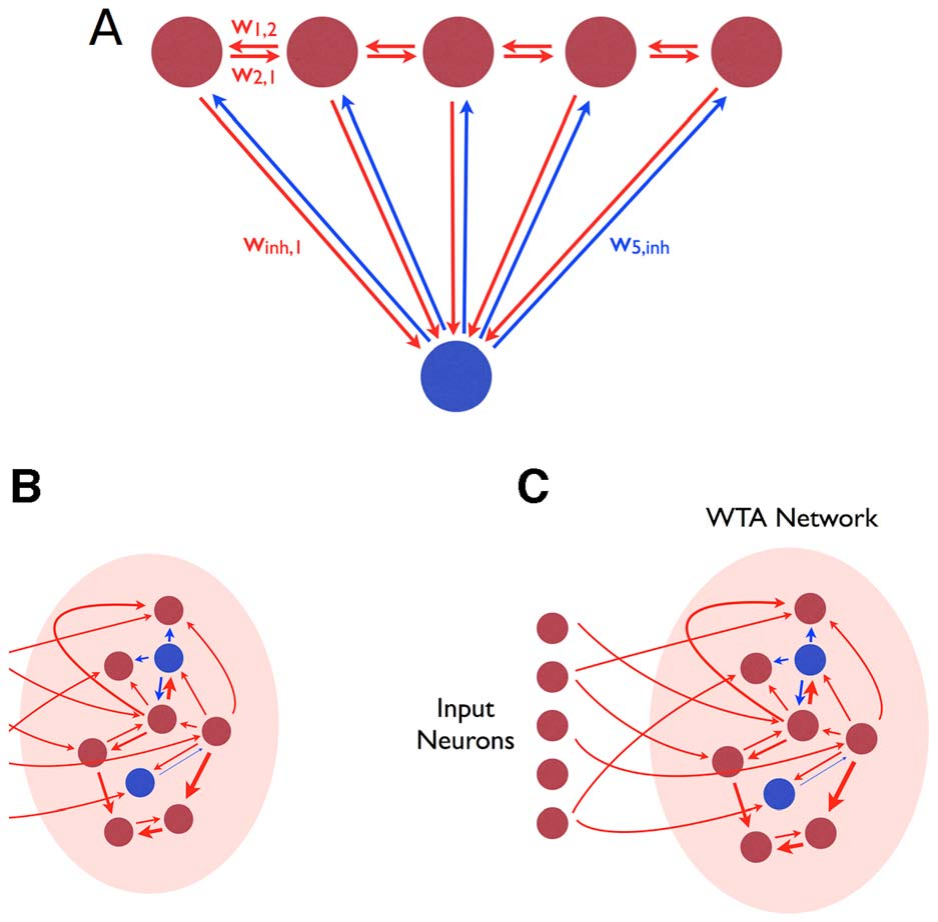
Winner-take-all architecture. (**A**) Architecture of an idealised winner-take-all-network. Several excitatory neurons (red) excite a single shared inhibitory neuron, or a shared population of inhibitory neurons (blue). Each excitatory neuron receives inhibitory feedback in proportion to the average activity of the excitatory population. (**B**) The WTA architecture is embedded in the field of recurrent connections between a population of excitatory and inhibitory neurons. (**C**) Once the WTA architecture has formed, coarsely structured synaptic input drives synaptic refinement of the recurrent connections within the network.

The idealized WTA network architecture is shown in Fig. 1A. Excitatory neurons are recurrently connected to each other and also with one or more inhibitory neurons, which project back to the excitatory neurons. This architecture does not in itself guarantee WTA functionality. The degree of recurrent excitation, excitation of inhibitory neurons, and inhibition of excitatory neurons need all to lie within preferred ranges [8] in order for the network to exhibit effective WTA behavior. The appropriate neural architecture must be grown, and then the weights of the many synapses must be tuned to fall within the necessary ranges. Such neuronal growth and synapse formation are subject to variability (1B,C), for which the homeostatic learning mechanisms must compensate.

The behavior of a WTA network depends on the ratios of the effects of its various excitatory and inhibitory connection paths. In its high excitatory gain regime a WTA network will report only the strongest of its feed-forward inputs, and suppress the remainder of the excitatory neurons, which are weakly activated. In a more relaxed regime (soft-WTA, sWTA) the network will return a pattern of winners that best conforms to its input. In this sense the sWTA performs a pattern based signal restoration, which is a crucial mechanism for resisting degradation of processing in neural systems across their many computational steps. In this paper we choose to have the developmental process grow and tune these sWTA networks.

Our goal is to demonstrate how plausible genetic developmental mechanisms can combine with homeostatic synaptic tuning to bring networks of neurons into sWTA functionality (Fig. 1). Our demonstration is based on simulations of the development and growth of neural tissue in 3D physical space using *Cx3D* [16]. The simulation begins with a single precursor cell. This cell encodes gene-like instructions that are sequentially and conditionally expressed through a gene regulatory network (GRN). By controlling the expression of different genes, this GRN gives rise to pools of differentiated excitatory and inhibitory neurons. These neurons, which are placed randomly in 3D space, extend axons and dendrites and make synapses according to a proximity rule. This process results in a synaptically connected network that matches well experimentally obtained connectivity statistics. During this neurite outgrowth, the synaptic weights calibrate themselves homeostatically using experimentally established synaptic scaling [17] and BCM learning rules [18]. This synaptic learning is conditioned by coarsely patterned neuronal activity similar to that of retinal waves or cortico-thalamic loops [19]–[23].

We compare these grown networks with biological data, and demonstrate WTA functionality. This comparison is done also in the context of cortical functionality, such as orientation selectivity. Importantly, the overall behavior stems solely from local processes, which are instantiated from internally encoded and developmental primitives [24]. Hence, we provide a model that explains the developmental self-construction and -configuration of a neocortical WTA network in a biologically plausible way.

## Results

### Development of Differentiated Neurons Based on a Gene-Regulatory-Network

Cell proliferation and differentiation into different cell types is specified implicitly in the genetic code of a single precursor cell. This code determines how a given number of excitatory and inhibitory neurons is produced. During the unfolding process of this code, each cell contains the same genetic code, but because of its local environment can follow different developmental trajectories.

We model the molecular mechanisms that regulate cell differentiation by a dynamical gene regulatory network (GRN). This GRN is defined by a set of 5 variables (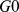, 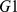, 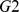, 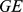, and 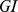) that represent substance concentrations, where each substance is the expression of a gene. Importantly, all cells have their own instantiations of these variables. The secretion, interaction, and decay of substances, is regulated by the laws of kinetics. The differential equations specifying these dynamics are shown in Methods.

During the evolution of the substance concentrations, also cell growth and division is simulated. The cell cycle time and model parameters of the differential equations are fixed and independent of the substance dynamics.

Initially, all concentrations are set to zero. At this stage, only the “starter” substance 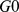 is produced, which reaches high concentration levels in the first time step, and triggers the production of a second gene 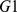. 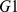 is produced according to a prespecified intrinsic production constant 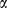. This value determines how many cell divisions will occur until the concentration of 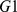 reaches a value of 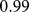. When this threshold is reached, a probabilistic decision is induced: 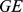 or 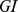, responsible for activating the excitatory and inhibitory cell phenotypes, are triggered with probability 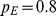 or 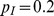, respectively.

**Figure 2:**
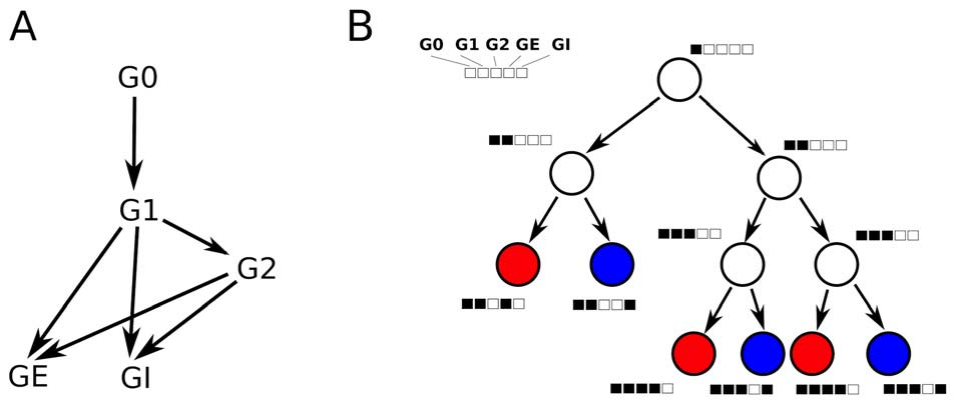
Gene Regulatory Network. (**A**) Schematic representation of the GRN, composed of five interacting genes that give rise to excitatory and inhibitory neurons. The identity of a neuron is determined by the genes GE and GI for excitatory or inhibitory neurons, respectively. Arrows indicate a positive effect on gene expression. (**B**) Lineage tree. Nodes indicate cells; boxes indicate gene expression patterns. G0 triggers the expression of G1, which characterizes the undifferentiated state of progenitor cells. After a series of symmetric divisions, G1 reaches a concentration threshold. According to fixed probabilities, G1 can then activate the differentiation toward excitatory (red) or inhibitory (blue) neurons. Alternatively, a small proportion of cells probabilistically undergoes a second round of cell division and activates gene G2, which again promotes the differentiation toward excitatory or inhibitory neurons by the expression of GE or GI. The probabilistic activation of inhibitory or excitatory genes is a simplification, but guarantees the production of a homogeneously mixed population of neurons.

Such a GRN network configuration would enable us to generate 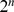 cells, where 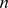 is the number of symmetric divisions. However, the target number of cells might not be an exponential of 2. Therefore, we have introduced a second gene 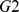 that is (probabilistically) activated by high concentrations of 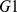, and that leads to a second round of symmetric division. As for 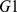, 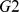 activates 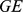 or 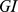 in a probabilistic manner. The probability to enter into this secondary cell cycle is given by 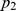, which is computed based on the target number of cells. The evolution of the GRN across cell types is depicted in Fig. 2.

By setting the production rate constant 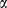 of gene 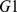 and the probabilistic activation of 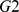, we can control the final number of cells produced. The equations for computing the probabilities for either differentiating into neurons by 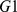 induction (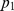) or by 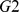 induction (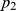), depending on the target number of cells, are shown in Methods.

**Figure 3:**
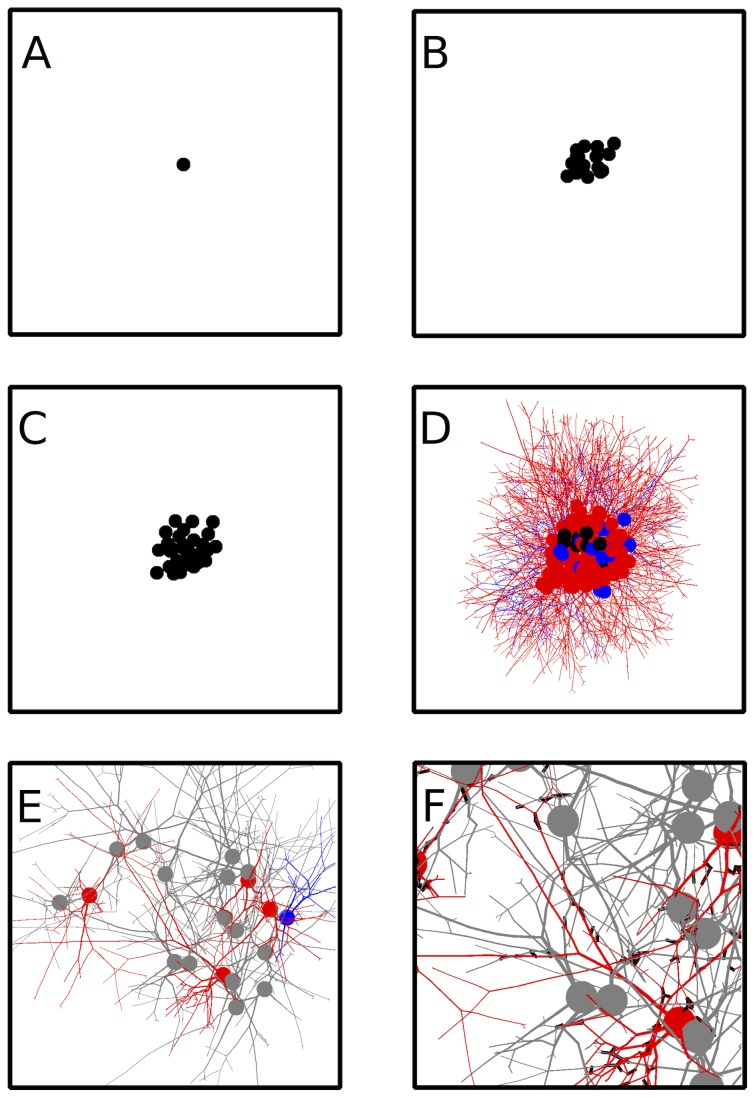
Developmental process for building a competitive network. A single precursor cell (**A**) contains the genetic code specifying the entire developmental process. (**B**) The precursor cell first undergoes repeated division to increase the pool of neuronal precursors (black). (**C**) Precursor neurons then differentiate into excitatory and inhibitory cell classes. (**D**) Neurite outgrowth begins to provide a scaffold for synaptic connections. (**E**) A network of differentiated neurons (grey) after neurite outgrowth has finished. For better visualization, examples of excitatory and inhibitory neurons are colored in red and blue, respectively. (**F**) Synapses (black rectangles) can form at appositions between axons and dendrites.

Overall, the GRN is designed so that a desired total number of cells is reached, and that the distribution of excitatory *vs.* inhibitory cells follows the approximate 4∶1 ratio observed in cortex [25]–[27] (S1 Figure). Fig. 3(A-D) shows the evolution of an initial cell giving rise to a number of cells which eventually grow out neurites based solely on their genetic encoding.

### Neurite Growth and Synapse Formation

Neurite growth and arborization is caused by growth cone traction and bifurcation. The growth cone is able to sense the presence and gradient of morphogens and other signal molecules, and also able to actively explore the local extracellular space. Importantly, neurite growth is steered via a growth cone model instantiated at the tip of the axon or dendrite, and so is a local process.

Diffusable signal molecules are secreted by the cell somata. In these simulations excitatory and inhibitory neurons secrete two characteristic signals, that enable excitatory and inhibitory axons to find inhibitory and excitatory neurons, respectively. The axonal growth cones initially grow out of the somata in random directions. However, they retract whenever the concentration they sense falls below a threshold. The retraction stops and growth recommences when a second higher threshold is exceeded. In this way the axons remain close to substance secreting sources. Retraction is an efficient strategy for establishing connections because axons grow only into regions containing a potential target, and is commonly observed in developing neurons [28]–[31]. A video of a developing neural network with axonal retraction (simulated in Cx3D) is included in the Supporting Information (S1 Video) and on Youtube (http://www.youtube.com/watch?v=il2uc-ZUZQ4).

Axons deploy boutons. Whenever these boutons are sufficiently close to a potential post-synaptic site on a dendrite a synapse is created between them. Consequently, the final synaptic network connectivity depends on the nearly stochastic arrangement of regions of spatial proximity of the outgrowing axons and dendrites.

**Figure 4:**
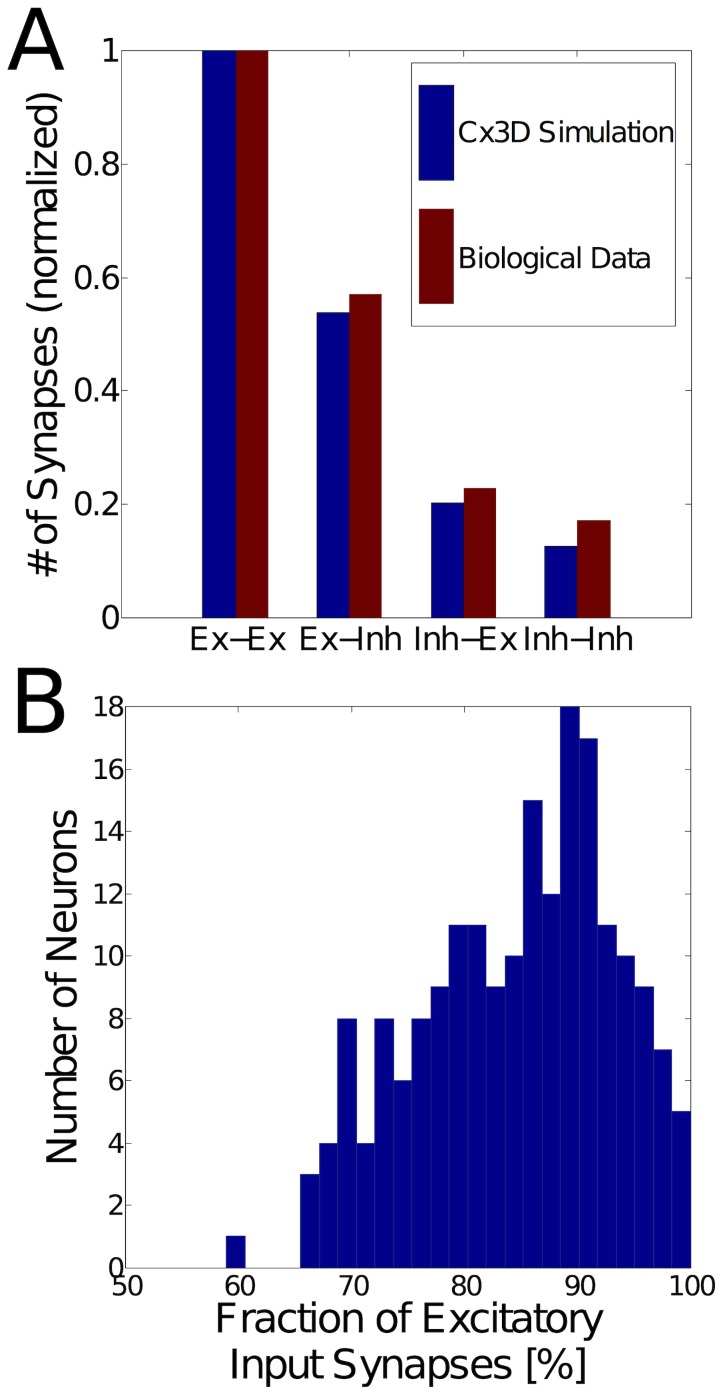
Connectivity after simulated neurite outgrowth. (**A**) Comparison of connectivity statistics from Cx3D simulations (blue) with experimental data (red) from [5]. Indicated on the vertical axis are the numbers (normalized with respect to the first bar) of synapses onto a single neuron. The individual bars show the values for the different pre- and postsynaptic neuron pairs (excitatory or inhibitory synapses onto an excitatory or inhibitory postsynaptic neuron). The numbers match in proportion, while the absolute quantities are higher in the biological data (approximately 155 *vs.* 3500 excitatory synapses onto a single excitatory neuron in the simulated and biological connectivity, respectively). This particular simulation consists of 250 neurons (200 excitatory and 50 inhibitory), which are randomly arranged in 3D space. (**B**) Histogram of the percentage of excitatory input synapses across the simulated network from (A). Each bar indicates the number of neurons that have a particular percentage of excitatory input synapses (after neurite growth and synapse formation have ended). The final distribution has a mean of 84%, which is in line with experimental assessments [5], [33]–[35].

We adapted the parameters of the neurite outgrowth (see Table 1) so that the connectivity of the simulated neuronal growth matched our experimental observations in layers II/III of cat visual cortex [5], [32] (see Fig. 4A). Overall, we found that connectivity was robust to reasonable variation of the growth parameters and the random location of somata. The absolute numbers of synapses simulated here are smaller than observed in biology, due to constraints on computational resources. However, there is no inherent restriction on scalability using our methods, and so we expect that realistic numbers of cells and synapses could if necessary be simulated using supercomputers.

**Table 1:** Parameters for simulating axonal and dendritic growth.

Parameter	Value (Ex. Axon/Inh. Axon/Ex. Dendrite/Inh. Dendrite)
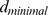 (minimal diameter)	0.2/0.2/0.3/0.3
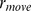 (diameter reduction when moving)	0.004/0.012/0.02/0.042
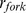 (diameter reduction when bifurcating)	0.12/0.105/0.14/0.12
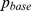 (baseline probability for bifurcation)	0.05/0.08/0.04/0.05
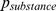 (substance dependent probability for bifurcation)	0.005/0.05/0.0/0.0
speed of growth	100/100/100/100
speed of retraction	5/5/(no retraction)/(no retraction)
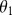 (concentration threshold triggering retraction)	1e-8/1e-8/(no retraction)/(no retraction)
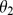 (concentration threshold stopping retraction)	0.036/0.036/(no retraction)/(no retraction)
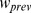 (weight of previous growth direction)	0.75/0.75/0.75/0.75
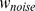 (weight of random direction)	0.25/0.25/0.25/0.25
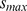 (neurite discretization size)	7/7/7/7

Growth parameters are dependent on the type of the neurite, as well as the neuron type. In order to qualitatively match biological observations, we modeled axons to be longer than dendrites and inhibitory (basket) cells to have smaller spatial extent than excitatory neurons [56], [132]–[134]. In our model, axons direct their growth based on extracellular substance concentrations.

Fig. 4B shows the distribution of the percentage of excitatory input synapses to the neurons, across the whole population. The average percentage of excitatory inputs to a neuron in this network is 84%, which is in good agreement with the experimental data. This result is consistent with observations across species and cortical areas that some 15% of all the synapses are GABAergic [5], [33]–[35], irrespective of neuronal densities. Importantly, this good agreement arises naturally out of the growth model, and did not require extensive tuning of the model parameters.

### Electrophysiology

The self-configuration of electrophysiological processing depends on the tuning of network synaptic weights and neuronal activity. In order to simulate this aspect of the developing networks, we must model also the electrical activity of neurons. However, the time scales of morphological growth and electrophysiological dynamics are many orders of magnitude different, and this difference makes for substantial technical problems in simulation.

For simplicity, and for minimizing computational demands we have used a rate-based approach to modeling neuronal activity. We approximate the neuronal activation by a linear-threshold function [36] that describes the output action potential discharge rate of the neuron as a function of its input. This type of neuronal activation function is a good approximation to experimental observations of the adapted current discharge relation of neurons [37], [38] and has been used in a wide range of modeling works [8], [39]–[41].

The linear-threshold activation function is:
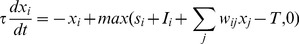
(1)where 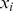 denotes the firing rate of a neuron with index i, 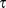 is the neuronal time constant, 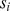 is the spontaneous activity, 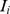 is the feed-forward input to neuron i, 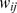 is the weight of the connection from neuron j to neuron i (can be positive or negative, depending on the presynaptic neuron's type), and 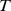 is the neuron's threshold. For simplicity, 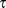 and 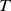 are set to 1 and 0. Exploratory simulations where 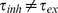 yielded very similar results.

For computational efficiency, the electrophysiology simulator is implemented as a global process that acts on the total weight matrix of the neuronal network, rather than performing these frequent computations locally. We chose this global methodology because it leads to a significant speed-up compared with a local version that had been used initially. The total weight matrix is obtained by summation of the weights of all synapses in the Cx3D simulation. Using these connection weights, neuronal activity is computed as described in Eq. 1. Connection weight changes resulting from the learning and adaptation (explained below) are computed based on this summed weight matrix and the activities of the two respective connected neurons, which are saved at each electrophysiology time step. The same connection weights (and neuronal activities) would be computed if only local processes at the synapses were simulated, because the synaptic learning and adaptation dynamics (Eq. 2 and 3) are dependent on the (locally available) neuronal activities, and linearly dependent on the synaptic weight. Hence, the dynamics of the summed synaptic weights match the sum of the individual synapse weight changes.

For reasons of biological plausibility, the electrophysiology simulator incorporates a maximum connection weight. This maximum weight for the functional connection strength between two neurons is determined by counting the number of synapses involved. This number, multiplied by the maximal weight of a single synapse, is defined as the maximum of the total connection weight. Hence, neurons that are connected by few synapses can not establish a strong functional link.

In our model, self-configuration of the weights towards sWTA functionality occurs during sequential developmental phases. Sequential phases of electrical adaptation and learning during development have been observed experimentally [42], [43], and have also been applied in previous models [44], [45].

During the first, homeostatic phase neurons adapt the synaptic weights of their own input in order to maintain a target output activity. The effect of this phase is to bring the neuronal firing rates into a balanced regime, and so allow for a reliable synaptic learning without interference by unresponsive neurons or run-away excitation. During the second, specification phase the neurons structure their individual responses by correlation-based learning on their inputs.

#### Homeostatic phase

During this first phase of activity-dependent adaptation, neurite outgrowth, synapse formation and homeostatic adaptation of neuronal activity occur simultaneously. Neurons implement the synaptic scaling rule [17], [46], [47], whereby they scale their synaptic input weights to achieve a preferred average output firing rate. Thus, when their average output activity exceeds a given target, neurons scale their excitatory and inhibitory inputs down and up respectively. The opposite effect occurs when the average activity has fallen below the target. Since there is no correlation-based learning during this phase the population of neurons can converge towards stable average levels of activity, but there is no input learning.

**Figure 5:**
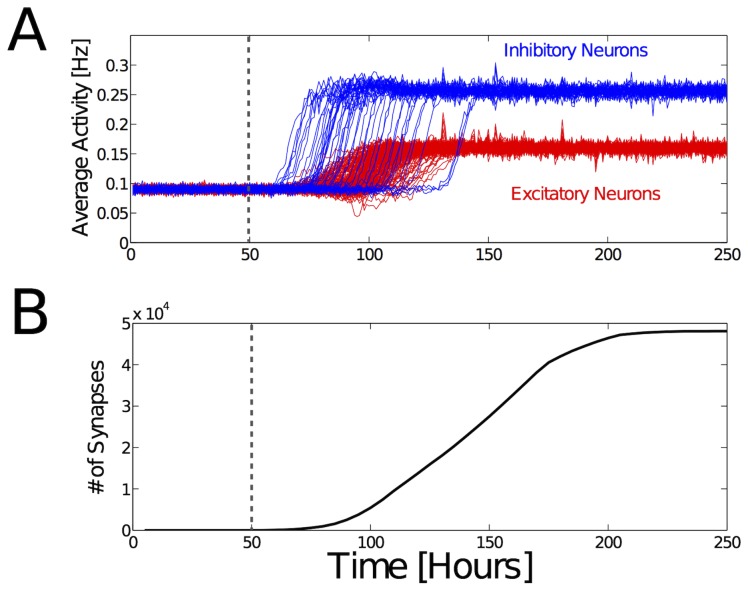
Homeostatic adaptation of neuronal firing rates during establishment of synaptic connectivity. (**A**) Synaptic scaling during neurite outgrowth leads to robust average activities of both excitatory (red) and inhibitory (blue) neurons. The network consists of 250 neurons that are randomly arranged in 3D space. The horizontal axis indicates the estimated real-time when taking into account that the time constant of synaptic scaling is in the order of several hours [17]. At 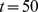 (dashed line), the neurite outgrowth begins. Average firing rates of layer II/III pyramidal neurons have been shown to be smaller than 1 Hz *in-vivo* [128], [129]. Experimental data indicates that inhibitory neurons have higher activities 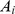 (Eq. 2) than excitatory neurons [68], [100], [130], [131]. In this simulation there are not yet any input projections, so the activity originates solely from internally generated and random activity. (**B**) Total (excitatory and inhibitory) number of synapses in the network during development. New synapses are formed also after the neurons reach the target average activities, without disrupting the homeostatic adaptation process or bringing the network out of balance. These simulation results demonstrate the robustness of the synaptic scaling process during network growth.

The equations for synaptic scaling are given by [48], [49]:
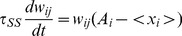
(2)where 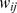 is the connection strength from neuron j to neuron i, 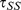 is the time constant of the learning rule (usually hours or days), 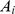 is desired average activity of postsynaptic neuron i, and 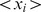 is the actual average activity of neuron i. Fig. 5 shows that this synaptic scaling permits the simulated network to reach a stable state with robust excitatory and inhibitory firing rates.

Post-synaptic scaling is not the only mechanism that can be used for neuronal activity homeostasis. For example, [49] has described an extended version of synaptic scaling: The presynaptic-dependent synaptic scaling (PSD) rule. We also implemented that PSD rule, but obtained results which differed only slightly from traditional synaptic scaling.

In the later stages of this first phase, input neurons (that are not part of the growing network) are added to the model (see Fig. 1C). These input neurons could correspond, for example, to thalamic or cortical layer IV neurons. They are initially fully connected to neurons of the grown network, and their projection efficacies are randomly drawn from a uniform distribution. Importantly, there is a neighborhood relationship amongst the input neurons: Input populations can be topologically close to, or distant from one another. The input neurons provide coarsely patterned input activity to the grown network. We chose hill-shaped patterns of activity centered on a given population, and decaying with topological distance from its center. The centers of these patterns move periodically in a noisy wave-like fashion (see Methods). This patterning of the electrical activity in the input layer can be interpreted as, for example, the retinal waves in early development [19]–[23], that can induce correlations within the activities of downstream neural subpopulation.

By the end of this homeostatic phase, neurons and synapses have reached their final structural configuration. Overall, this phase prepares the network for the next phase of correlation-based learning of input stimuli.

#### Specification phase

In this phase synapses onto excitatory postsynaptic neurons obey the Bienenstock-Cooper-Munro (BCM) learning rule [18], [50], [51], rather than synaptic scaling. The BCM rule is composed of a Hebbian term, and a homeostatic term which determines whether the Hebbian synapse grows stronger or weaker.

The BCM learning rule is:
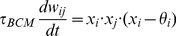
(3)


where 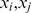 denote the discharge rates of post- and pre-synaptic neurons 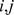; 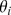 is the averaged square of neuron 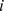
's firing rate, multiplied by a constant (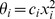). The constant 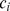 determines the average firing rate that the neuron converges towards in the stationary state; the condition 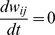 is met in the non-trivial case where 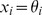. Let 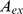 and 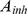 denote the target average firing rates of excitatory and inhibitory neurons, respectively. Then in order for the neurons to converge to these firing rates, 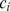 is set to 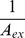 if neuron 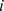 is excitatory, or 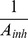 if it is inhibitory.

All synapses (excitatory and inhibitory) made onto excitatory neurons follow the BCM learning rule, while those onto inhibitory neurons follow the synaptic scaling (Eq. 2) rule. While learning is commonly attributed to excitatory synapses, inhibitory synapses can also undergo long-term potentiation (LTP) as well as long-term depression (LTD) [52]–[55].

The lack of a correlative term for synapses onto inhibitory postsynaptic neurons is, as shown below, necessary to match experimental data on orientation selectivity of excitatory and inhibitory neurons in mouse visual cortex. We therefore hypothesize that basket cells in the superficial layers of cortex homeostatically adapt their input synapses, in contrast to pyramidal neurons, which also use correlational information.

We have also explored the case in which the same learning rule is used by all synapses. This case also yields WTA functionality (see below). Given that there are many different classes of inhibitory neurons [56], which differ also in their developmental characteristics [57], it is possible that different interneuron types follow different learning rules.

### Functional Properties

#### Self-organization of WTA functionality

**Figure 6:**
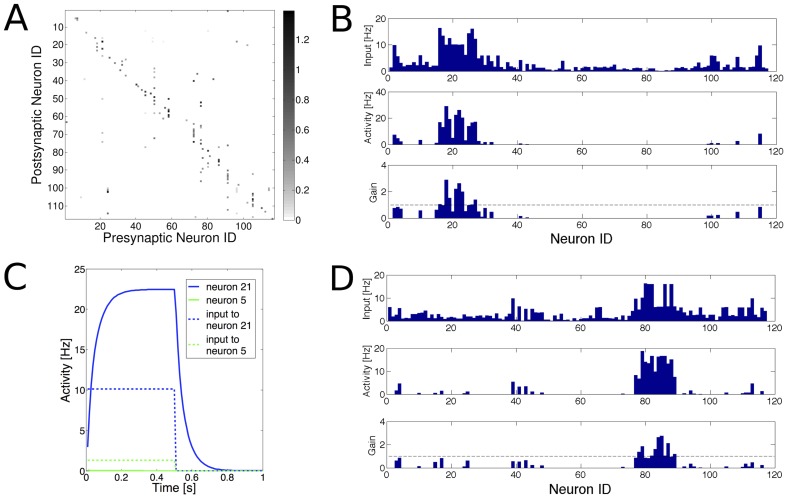
Winner-take-all functionality. (**A**) Weight matrix of 117 excitatory neurons in a WTA network. After learning the network exhibits a 1-dimensional neighborhood topology, as shown by the predominantly strong weights around the diagonal. This topology mirrors the neighborhood relationship of the input stimuli, which are continuously and periodically moving hills of activity. Only the excitatory connections are shown here, because the inhibitory neurons do not integrate into the neighborhood topology (see text). (**B**) Demonstration of WTA functionality on the network connectivity shown in (A). Neurons are ordered here such that adjacent neurons connect most strongly. The input to the network (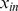; top row) has a hill shape, with added noise. The network response (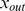; middle row) is a de-noised version of the input with the bump in the same location. The neuronal gain (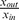; bottom row) is high for neurons receiving the strongest input, and low (or zero) for neurons distant from the main input to the network. The dashed horizontal line indicates a gain of 1. (**C**) Activity of a winning neuron (blue, solid), during presentation of its feedforward input (blue, dashed) in the same simulation as shown in (B). Recurrent connectivity amplifies the response of the neuron for the duration of the stimulus (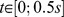). In contrast, a losing neuron (green, solid) receives non-zero feedforward input (green, dashed), but is suppressed due to the WTA functionality of the network. (**D**) Response of the same network to a different feedforward input. The recovery of a bump shaped activity can occur anywhere in the network topology.

As a consequence of the synaptic learning in the second developmental phase, the network learns the topology of its inputs. Those neurons which are excited by a common input, become more strongly connected with one another. Because of the competition that is inherent to the BCM learning rule, excitatory neurons become progressively more connected to only particular input neurons (those which evoke their strongest response), while decreasing their affinity to the others. Fig. 6A shows that the final functional connectivity of excitatory neurons indeed exhibits a strong neighborhood relationship: The connection weights are stronger around the diagonal, so that the neurons are close to or distant from one another in weight space. This connection topology reflects the (1-dimensional) topology of the input patterns.

The inhibitory neurons do not integrate into this topology because the synapses onto inhibitory neurons follow the non-Hebbian synaptic scaling rule, and so their input correlations can not be learned. Fig. 6B,D show examples of the final soft-WTA functionality, after the network has learned the input topology. The excitatory neurons receiving the largest input are predominantly enhanced due to recurrent excitation with one another. The inhibitory neurons reflect the overall activity, and reduce the losing neurons' activity, more than they are enhanced by excitatory inputs. From a functional point of view, this active selection of the winning population improves the signal to noise ratio, and confirms their sWTA properties.

#### Unsupervised clustering

**Figure 7:**
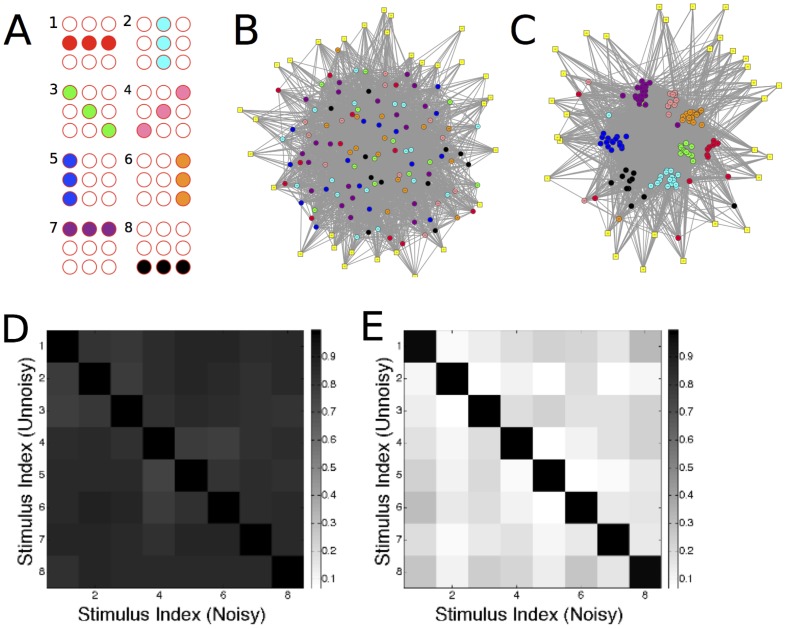
Clustering and decorrelation of representations. (**A–C**) Discrete input patterns give rise to clusters in the functional connectivity of the WTA network. (**A**) Input stimuli used in the learning process. Filled and empty spheres indicate strongly and weakly active populations, respectively. (**B,C**) Visualization of the network structure before and after learning. Strongly-coupled neurons are drawn close together; excitatory synaptic connections are indicated by grey links. Excitatory neurons are coloured according to their preferred input pattern (colours in A); inhibitory neurons (square) are drawn in yellow. (**B**) Before learning, no clustering of synaptic connections is present. (**C**) After learning, neurons with the same preferred stimulus are strongly interconnected. See S2 Video. (**D**) Before learning, the response of the network is similar across all stimuli. Shown is the scalar product between the vectors of neuronal responses to pairs of stimuli 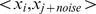. The noise was added in order to assess the sensitivity of the network's activity to a perturbation of the input signal (see text). The high values and uniformity of scalar products in (D) indicates that network responses poorly distinguish between stimuli. (**E**) After learning, responses to noisy stimulus presentations are highly similar (high values of scalar product; black diagonal), whereas responses to different stimuli are decorrelated (low values of scalar product; light shading).

WTA networks are able to perform pattern recognition and classification, i.e. that neurons cluster functionally and respond to patterns in a discriminative and classifying manner. We explored whether this property can arise in a biological setting, as captured by our developmental model. To do this, the processes of connectivity establishment and synaptic homeostasis were simulated as described before. However, during the learning phase input patterns consisting of discrete bars of different position and orientation (Fig. 7A) were presented to the network. In this input regime there are no continuous orderings between individual patterns (which is the case for the retinal-wave like activation patterns).

Learning the discrete input stimuli causes the population to partition into sub-populations, or assemblies, as shown in Fig. 7C. We demonstrated the generality of this learning by simulating the clustering in response to presentation of only 4 input stimuli, using the same network and simulation parameters as in the case with a full range of stimuli (S2A Figure). We also examined the scenario in which all synapses (including those onto inhibitory neurons) follow the same BCM learning rule. As anticipated, this case yielded networks in which inhibitory neurons cluster along with the excitatory populations (compare S2B Figure with Fig. 7C).

The clustered functional connectivity allows the network to decorrelate its inputs, so that even noisy signals can be reliably differentiated. We quantified this ability by testing the network response to a particular pattern 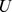, by comparison with pattern 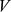. This comparison was measured using the scalar product between the activities in the network after presentation of the different input patterns. Let 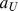 and 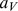 be the n-dimensional vectors of the neuronal activities in a network of n neurons, in response to the stimuli 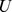 and 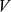, respectively. The scalar product 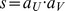 then quantifies whether the responses to the two stimuli 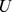 and 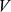 are very different (s close to 0) or correlated (s close to 1). To demonstrate that the results are valid under more biologically plausible conditions, noisy stimuli were used. A noisy input stimulus is defined as:
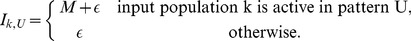
(4)


where k is the index of the input population and U is the stimulus identifier. M is the amplitude of the active populations in the input (which we set to 10 Hz in this case), and 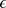 is uniformly distributed noise in the range 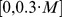. Fig. 6D, E shows the correlations of the network's responses for 8 different input stimuli before (D) and after (E) learning under noisy *vs.* not noisy (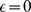) conditions. The off-diagonal elements in the correlation matrix are much lower after learning than before. These results demonstrate the decorrelation of the network's activity, and the robustness to input noise.

#### Competition between states

**Figure 8:**
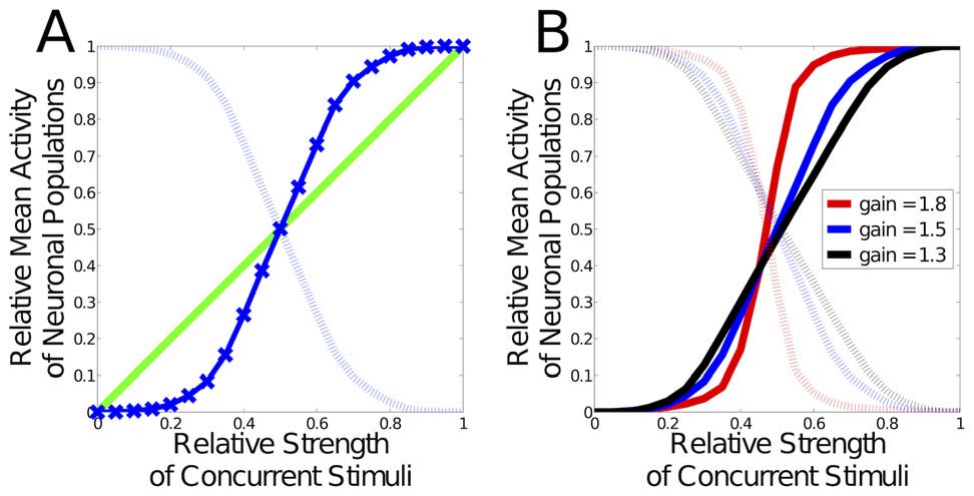
Stimuli are represented by competing subpopulations. (**A**) Competition for representation of a mixture of 2 concurrent stimuli. Shown is the normalized average activity of two sub-populations, in response to mixtures of the preferred stimuli of the two populations. For mixtures containing predominately one stimulus (mixture proportions close to 0 and 1), the populations are strongly in competition, and the network represents exclusively the stronger of the two stimuli (responses near 0 and 1). For intermediate mixture proportions, competition causes a rapid shift between representations of the two stimuli (deviation from diagonal reference line). (**B**) Increasing the gain of the network 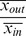 (black line: 1.3, blue: 1.5, red: 1.8) increases the stability of representations, and increases the rate of switching between representations due to stronger competition.

In addition to the decorrelation of responses, clustering provides competition between inputs. This property is computationally interesting because it forces the network to make a decision based on its input (Fig. 8A). We demonstrated this competition by presenting simultaneously two competing patterns (after the network had learned 4 different patterns). The relative proportion of these patterns in the input was gradually varied between the first and second pattern.

The results show that the stronger stimulus non-linearly dominates responses in the WTA network, so that the masked stimulus evokes an activity pattern that resembles that evoked by the strong stimulus alone. These results are in accord with experimental studies in visual cortex [58]–[60] and auditory cortex [61].

The nature of the competition between the states is dependent on the functional connectivity. Strong recurrent excitation (i.e. a high gain) yields strong inhibition, which results in a marked switching behavior between the different populations. This is because the competition is strong, and so the switch from one state to the other is more evident. A high slope of the transition reflects a functionality similar to a bistable switch. More specifically, the slope of the transition (middle part of the interpolation in Fig. 8A) increases with the gain of the WTA network. This gain can be adjusted via the homeostatic average activities: Higher target activities lead to more recurrent excitation, which increases the gain. Such differently graded competition is seen in Fig. 8B. Bistability is also interesting from a computational point of view, because discrete states can be represented reliably. This kind of reliability is useful for performing computation with states based on analog elements [8], [62]. Competition also develops when synapses onto excitatory as well as inhibitory neurons follow the BCM learning rule, as shown in S3 Figure.

### Correspondence with Orientation Selectivity of Excitatory and Inhibitory Neurons

**Figure 9:**
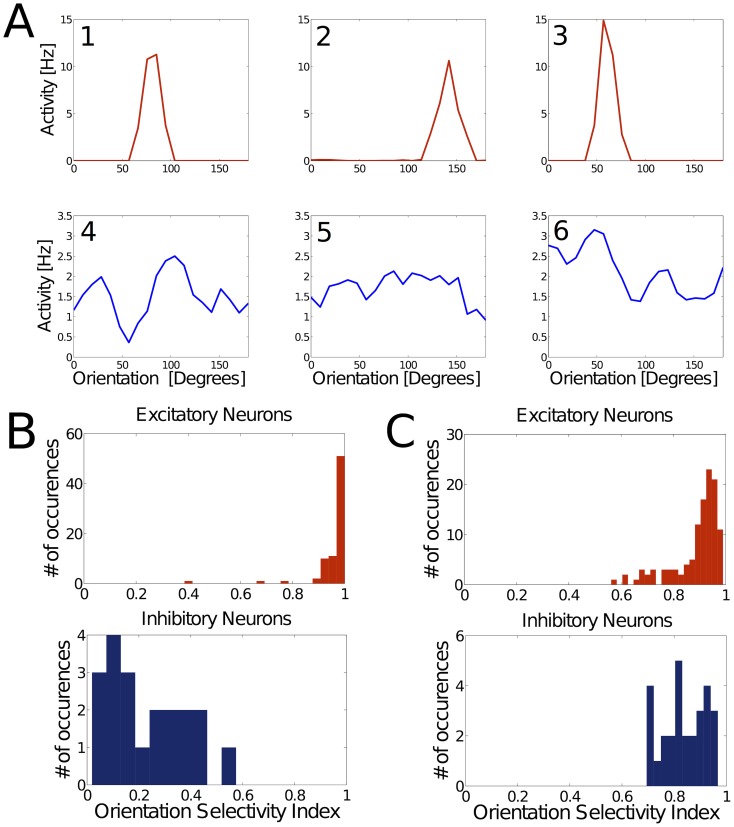
Excitatory neurons are strongly tuned; inhibitory neurons are poorly tuned. Tuning properties of excitatory and inhibitory neurons. (**A**) Representative tuning curves for 3 excitatory (red, 1-3) and 3 inhibitory (blue, 4-6) neurons in a WTA network after the learning process. Excitatory neurons exhibit strong and narrowly tuned preference for certain inputs, in contrast to inhibitory neurons. (**B**) Distribution of the orientation selectivity index (OSI) across all excitatory and inhibitory neurons in a WTA network, demonstrating the discrepancy of tuning on a population level. (**C**) Simulation of the same learning rule for synapses onto excitatory as well as inhibitory neurons yields orientation-tuned neurons in both populations.

We investigated whether our developmental model can account for experimental findings on orientation selectivity in visual cortex; for example, differences in tuning between excitatory and inhibitory neurons. In order to address this question, we assumed that the hills of activity in the input layer correspond to oriented stimuli (e.g. bars), which are smoothly and periodically rotating between 0 and 180 degrees. As anticipated from the previous results, excitatory neurons become highly orientation selective (Fig. 9), in contrast to inhibitory neurons. These results are in line with biological data. For example, [63] have analyzed orientation selectivity of excitatory and inhibitory neurons in mouse visual cortex. They report inhibitory neurons to be more broadly tuned and hence less selective than excitatory, pyramidal neurons. Similar findings were reported by [64]–[68].

We also quantified the orientation tuning based on the orientation selectivity index (OSI), which specifies the degree to which a neuron is selective for orientation. The value of this index lies between 0 (non-selective) and 1 (selective to a single, specific orientation). Fig. 9B shows the distribution of the OSI for excitatory and inhibitory neurons in a WTA network, demonstrating the discrepancy of orientation selectivity also on a population level. We conducted additional simulations, which demonstrated that when inhibitory neurons follow the same learning rule as excitatory neurons, they exhibit more narrowly tuned orientation selectivity (Fig. 9C). Hence, experimental findings of orientation selective inhibitory neurons in cat visual cortex [69]–[72] can also be accounted for by our model.

### Inhibition of Excitatory Neurons

We have analyzed the consequences of our model on the nature of the inhibition of excitatory neurons. As mentioned above, inhibitory synapses onto excitatory neurons are subject to the BCM learning rule (Eq. 3).

The competition between excitatory neurons depends on the common input that they all receive from inhibitory neurons. This common input must reflect the overall activity of the network, so that the competition is suitably normalized. However, the inhibition of the excitatory neurons stems from multiple inhibitory neurons, which should partition their common inhibitory task amongst each other in a self-organizing way. We investigated this partitioning, and how an excitatory neuron is inhibited during stimulation.

In order to quantify the impact of a neuron j on another neuron i for a given stimulus, we calculate a value that we will call the recursively effective exertion (REE). It is obtained by multiplying the activity of neuron j (under a given stimulus 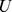) with the total connection weight 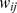 from neuron j to i:
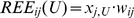
(5)


**Figure 10:**
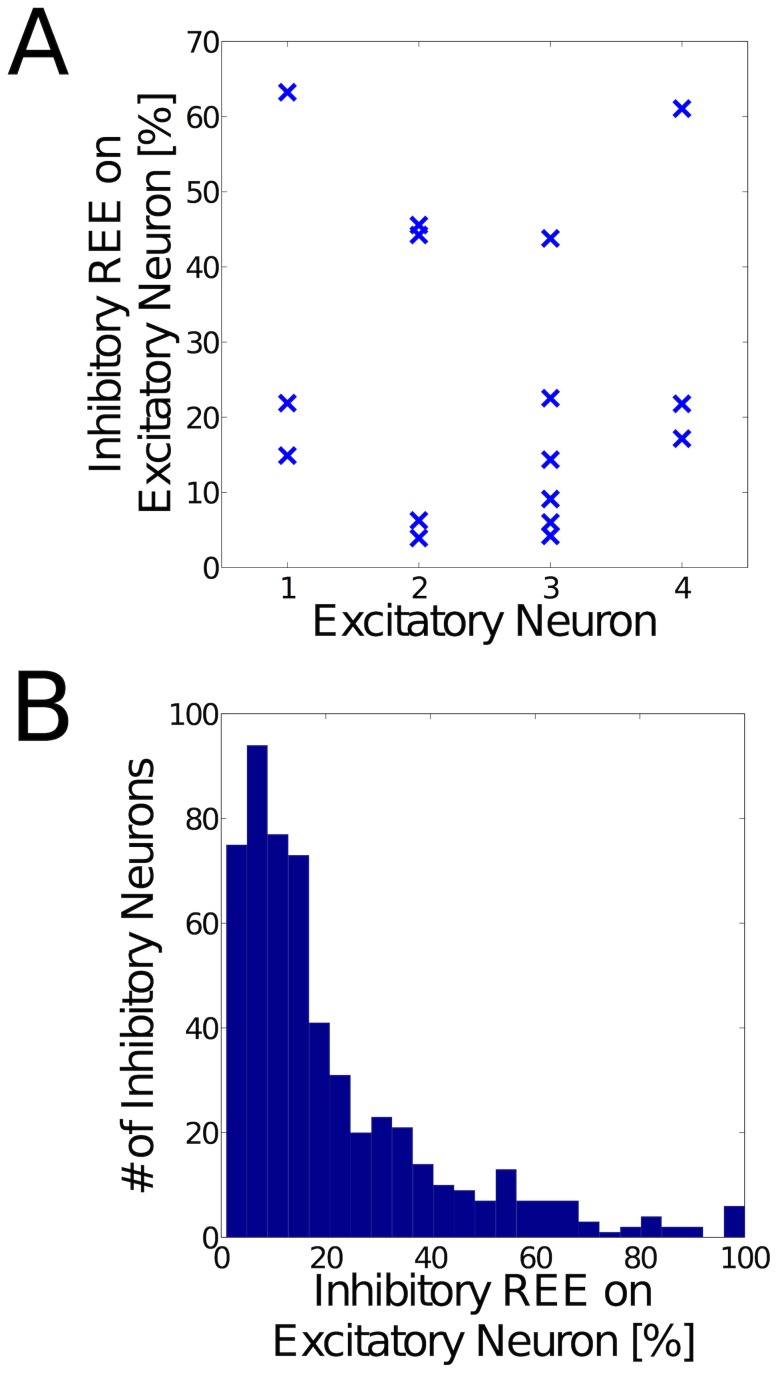
Inhibition of excitatory neurons. Excitatory neurons are predominantly inhibited by subsets of the inhibitory neurons that project to them. (**A**) Representative examples of the inhibition to excitatory neurons in a learned WTA network, during presentation of a stimulus. The vertical axis indicates the percentage of the total inhibitory REE (see definition of REE in text) that an individual inhibitory neuron delivers to this particular excitatory neuron. Few (usually 2 or 3) inhibitory neurons provide the major part of the inhibition. (**B**) Histogram of all the REE contributions (in %) from inhibitory neurons, across all excitatory neurons in the WTA network. The distribution shows that few inhibitory neurons provide the major part of the inhibitory REE on an excitatory neuron. This specialization is a result of the BCM learning rule, which is followed also by inhibitory synapses onto excitatory neurons.

The REE value is therefore stimulus-dependent, and dependent on the recurrent network connectivity. Fig. 10 shows that inhibition is distributed non-uniformly: A few inhibitory neurons dominate the suppression of an excitatory neuron. This dominance is due to the BCM learning by inhibitory synapses: Strongly and weakly correlated inhibitory connections to excitatory neurons are strengthened or weakened, respectively. These inhibitory connection strengths converge because of the homeostatic activity regulation, which is part of the BCM learning rule.

The nature of inhibition of excitatory neurons is interesting in the context of the anatomy of inhibitory basket cells. These neurons predominantly target locations close to the soma or the proximal dendrites, where they can strongly influence the excitatory neuron [73]. Therefore, it is plausible that the recruitment of a small number of inhibitory neurons is sufficient to inhibit an excitatory neuron. Electrophysiological experiments could in principle validate this hypothesis by showing that only a small proportion of the inhibitory neurons projecting to a pyramidal neuron are predominantly responsible for its suppression.

## Discussion

In this paper we have demonstrated by simulation of physical development in a 3D space, how an autonomous gene regulatory network can orchestrate the self-construction and -calibration of a field of soft-WTA neural networks, able to perform pattern restoration and classification on their input signals. The importance of this result is that it demonstrates in a systematic and principled way how genetic information contained in a single precursor cell can unfold into a functional network of neurons with highly organized connections and synaptic weights.

The principles of morphological and functional development captured in our model are necessarily simplified with respect to the boundless detail of biology. Nevertheless, these principles are both strongly supported by experimental data, and sufficiently rich in their collective expression to explain coherently the complex process of expansion of a genotype to a functional phenotypic neuronal circuit. In this way our work offers a significant advance over previous biological and modeling studies which have focused either on elements of neuronal development, or on learning in networks whose initial connectivity is given. Therefore we expect that methods and results of the kind reported here will be of interest both to developmental biologists seeking a modeling approach to exploring system level processes, as well as to neuronal learning theorists who usually neglect the genetic-developmental and homeostatic aspects of detailed learning in favor of an initial network that serves as a basic scaffold for subsequent learning [74]–[76].

It is relatively easy to express a well-characterized biological process through an explicit simulation. That is, one in which the simulation simply recapitulates the process by expanding some data through a simple model, without regard for physical and mechanistic constraints. By contrast, the simulation methods [16] that we have used here are strictly committed to physical realities such as 3D space, forces, diffusion, gene-expression networks, cellular growth mechanisms, etc. Our methods are also committed to local agency: All active processes are localized to cells, can only have local actions, and have access to only local signals. There is no global controller with global knowledge, able simply to paint the developmental picture into a 3D space. Instead, the ability of a precursor cell to expand to a functional network is the result of collective interaction between localized cellular processes. And overall, the developmental process is the expression of an organization that is encoded only implicitly, rather than explicity, in the GRN of the precursor cell. Thus, our GRN encodes constraints and methods rather than explicit behaviors.

In previous work [77], [78] we have shown how this approach can be used to explain the development of neocortical lamination and connectivity. In that case we did not consider also the electrophysiological signaling between cells and so the self-configuration of their computational roles, as we have done here. However, the incorporation of electrophysiological signaling into the growth model brings substantial technical difficulties, such as those arising out of the large differences in spatio-temporal scales between cellular developmental and electrophysiological signaling processes, as well as the supply and management of sufficient computational resources. Therefore we have chosen to keep these problems tractable in this first functional study, by restricting our question to a sub-domain of cortical development: How could neuronal precursors expand into functional circuits, at all. Even then, we must be satisfied for the moment with a rate based model of neuronal activity, rather than a fully spiking one.

The emphasis of this paper is on the process whereby a precursor expands to some useful network function. The particular function is less relevant, and in any case the functional/computational details of cortical circuits are as yet not fully understood. We have chosen to induce WTA-like function because our previous work has been focused on the likely similarity between the WTA motif and the neuronal types and their inter-connectivity in the superficial layers of cortex [6]. Moreover these WTA networks are intriguing from both the biological, and computational perspective [3], [6]–[15], [41]. The strong recurrent excitation available in the superficial layers of cortex, and their critical dependence on feedback inhibition has been clearly demonstrated by intracellular recordings in the presence of ionophoretic manipulation of GABA agonists and antagonists [4]. These relationships are crucial for WTA-like processing, because they offer the network induced gain that is crucial for providing the signal restoration, signal selection, and process control that support systematic computation. Recent optogenetic studies appear to confirm the presence of circuit induced gain, in the input layers of mouse visual cortex [79], [80]. Taken together these experimental and theoretical results support the hypothesis that at least some fundamental WTA functionality is embedded in the processing architecture of superficial neuronal circuits, and so makes the WTA motif a worth target of the developmental process that we have described here.

Our model predicts that neurons form specific subgroups, or cell assemblies [81], [82]. There is indeed strong evidence from biological data for this clustered connectivity [83]–[85], which (as in our simulations), appears to be grounded in the similarity of functional selectivity [86].

We did not allow dynamic rearrangement of synapses in these first simulations. However, it is plausible that weak synapses are pruned away, freeing synaptic resource to explore for more correlated partners.

Peters' rule [87]–[89] proposes that connectivity can be estimated by the product of the random overlap of pre- and postsynaptic sites. This rule may be true for average connectivity, but specific functionality obviously calls for more specific low level connectivity within the average. One opinion is that such specificity is explicitly genetic, and so accounts for example for the diversity of cortical interneurons [90], [91]. Instead, our result speaks for an implicit rather than explicit genetic specificity. That is, the apparently specific wiring of the WTA network arises by neurons collectively satisfying genetically expressed constraints. This concept is in stark contrast to the view that network functionality emerges from individual processes that do not coordinate with potential interaction partners. In our simulations, a neuron's morphology and the functional strengths of its synapses depend on the collective behavior of the other neurons. Hence, the structure and function of a neuron grown in isolation is different from a neuron with the same genetic code, but that interacts and coordinates with its environment during development.

Our learning rule requires that input projections are ordered in such a way that their collective input patterns provide at least a coarsely structured signal against which the presumptive WTA layer of neurons can successfully deploy a BCM-like learning mechanism. This ordering is not a stringent requirement. For example, provided that there is some degree of coherent axonal mapping of axons from input neurons of the subplate or thalamus into the target WTA layer, then even metabolically induced travelling waves of activity across the developing input population could provide a sufficiently structured signal for learning.

Traditionally, many modeling studies have been based on the assumption that the limited lateral extent of the neuronal axonal and dendritic tree naturally leads to a properly configured 2D neighborhood topology [92]–[94]. However, it is unclear how more realistic anatomical properties (anisotropy, variation of neurite extent, irregular locations of somata etc.) affect these topologies. Our work addresses this problem by demonstrating how neurons can self-calibrate in a stimulus-induced way, within a non-uniform and irregular neuronal setting. Hence, our work provides a better understanding of how developmental mechanisms can generate a neighborhood topology, and so is complementary to the classical approach.

As development of input neurons proceeds, the degree of structuring is likely to improve also, so that input neurons projecting to the same targets share similar features (for example, their ON- and OFF-subfields). This is in line with studies on thalamo-cortical projections [95], as well as cortico-cortical projections from layer IV to II/III [96]. However, it should be noted that this input specificity does not play onto inhibitory targets, which is in accordance with our work. Since the input to the neurons shapes the functional connectivity in the network, it follows from our model that neurons which receive common input are more likely to connect with each other (assuming that structural connectivity is adjusting to functional connectivity). The studies of [96] and [97] provide evidence for this input-dependent intra-network specificity.

Our results predict that only a few inhibitory neurons provide the major part of WTA-relevant inhibition, i.e. a relatively small subset of all the inhibitory basket cells projecting to a single pyramidal cell is responsible for its WTA suppression. These results suggest that WTA inhibition might not be very redundant, so that de-activation of only a few inhibitory neurons could result in very different electrophysiological behavior of pyramidal cells.

Our networks employ Hebbian-type learning for both excitatory and inhibitory synapses onto excitatory postsynaptic neurons. It is known that inhibitory synapses can undergo long-term potentiation (LTP) as well as long-term depression (LTD) [52]–[54], and learning by inhibitory synapses has been used in previous modeling studies [45], [98].

Non-Hebbian synaptic scaling of synapses onto inhibitory neurons results in orientation-nonselective inhibitory neurons. This distinction with respect to pyramidal neurons has been observed in mouse visual cortex, where the tuning of inhibitory neurons is broader than that of excitatory neurons [63]–[67], [99]–[101]. There is evidence for broadly tuned thalamo-cortical input to inhibitory neurons [95], as well as cortico-cortical input to those of layer II/III of mouse cortex [100]. Therefore we propose that at least some types of inhibitory neurons (e.g. fast-spiking (FS), PV-expressing interneurons) do not selectively adjust their inputs, but uniformly adapt the electrophysiological properties of their inputs for homeostasis.

Orientation-selective inhibitory neurons are found in cat visual cortex [69]–[72]. Since we do not model orientation maps, our findings are not directly applicable to the cat. However, we argue that it is the spatial location in the orientation map that determines the tuning curve of inhibitory neurons. Most cortical interneurons have a small horizontal dendritic extent [56], and so they likely receive inputs from similarly tuned excitatory neurons within an orientation map. Inhibitory neurons located close to orientation pinwheels are expected to have relatively broad orientation tuning, as reported in the above studies. The unbiased pooling of surrounding activity by inhibitory neurons is also supported by experimental results across species and sensory modalities [63], [101]. By contrast, we have shown that inhibitory neurons become orientation-selective when they follow the same (BCM) learning rule as excitatory neurons.

Our learning model provides a computational explanation for why most interneurons are smooth, i.e. have very few dendritic spines. It is believed that spines, by compartmentalizing biochemical signals, provide the molecular isolation required for independent synaptic learning [102]–[104]. The nonspecific and homogeneous adaptation of inhibitory neurons, which in our model are homogeneously scaling the input efficacies, is therefore well in line with this suggested function of dendritic spines. This model also provides an explanation for the finding that inhibitory, but not excitatory neurons exhibit structural remodeling of dendrites in the adult rat [105]. Changes in excitatory morphology at the level of dendritic branches (rather then spines) could have detrimental effects on already consolidated memories. Inhibitory neurons may retain their potential for dendritic restructuring, because their homeostatic adaptation does not interfere with learning of sensory experience.

We believe our findings to be robust also with respect to models incorporating spikes, because the main features of the adaptation and learning behavior have been demonstrated also on this more detailed level of electrophysiology. Along these lines, the studies of [106], [107] have explored spike-based WTA network functionality. Spike-dependent plasticity (STDP) is a Hebbian learning rule [108] and can yield synaptic homeostasis [109]. In particular, the BCM learning rule has been related to STDP mechanisms [109]–[112].

The robust self-organization of the WTA network is remarkable in that it arises out a single precursor cell, by simple genetically encoded rules. In future, this genetic developmental approach to functional circuit construction could be extended to larger networks composed of multiple WTA networks. For example, it has been hypothesized that by cooperation of multiple WTA circuits, the superficial layers of cortex could perform context-dependent processing [8]. Along these lines, [78] provide a model for the development of long-range projections connecting multiple columns, arranged on an hexagonal grid, as is observed in the superficial patch system [113]–[116]. It also remains to integrate these computational aspects into the context of a laminated cortical structure, which has already been simulated in Cx3D [24], [77].

## Materials and Methods

### Simulation

#### Cx3Dp

The growth simulations were conducted with the open-source package Cx3Dp, the parallelized version of Cx3D [16] (available at http://www.ini.uzh.ch/projects/cx3d/). As in the non-parallel version, neurons in Cx3Dp are decomposed into discrete spherical or cylindrical *physical elements* emulating the physical properties of developing tissues, whereas the biological properties derive from *modules*, that is, smaller programs expressed within specific physical elements. This local instantiation forces simulations to be based only on local interactions, without any global control of the developmental processes.

The default parameters for physical objects in Cx3D were initially chosen so that (1) density of cells/neurites can not be infinite, (2) objects do have a minimal adhesive property ensuring tissue integrity and (3) viscosity and not mass opposes to movement (see [16]). For the present study, we did not have to modify these default parameters.

#### Computer specifications

We used a rack computer with two 12-core AMD Opteron 6168 processors (1.9 GHz, 64 GB of RAM), running under Ubuntu 12.04 LTS.

### Gene Regulatory Network (GRN)

The GRN is defined by a set of variables 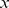 that represent genes and the corresponding substance concentrations. Changes in substance concentration are described by the rate equation:
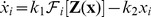
(6)


where 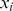 is the concentration of a protein encoded by the gene 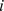 (i.e. 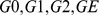 or 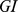), and 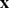 the corresponding concentration vector. The function 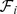 expresses how the synthesis rate of the protein encoded by gene 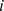 depends on the cooperative binding of all the substances, and 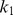, 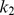 represent the production and degradation rates (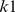, 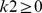). 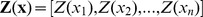 is a vector of Hill functions, which compute the binding probability of a substance 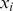 to a regulatory region given the affinity constant 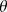, cooperativity 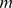 and binding bias 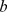:
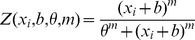
(7)


Gene substances can regulate gene expression by binding to specific sites in the genomic cis-regulatory regions. Substances that regulate each others' transcription are called transcription factors. Many genes are controlled by a number of different transcription factors and different arrangements of binding sites can compute logic operations on multiple inputs. Here, the function 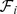 takes the form of a logical combination of interacting substances and is defined by the elementary operations:
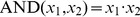
(8)

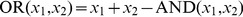
(9)

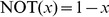
(10)More information on this description of GRN dynamics can be found in [117], [118]. Although abstract, this formalism can be directly translated into the corresponding mechanistic, kinetic differential equations. For our computational model based on 5 genes, we have used the following equations:
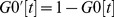
(11)

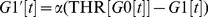
(12)

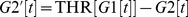
(13)

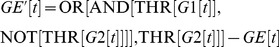
(14)

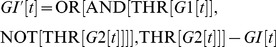
(15)


with:
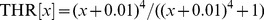
(16)


The probabilities of either differentiating into neurons by 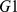 induction (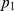) or by 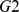 induction (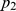) are computed as follows:
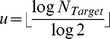
(17)

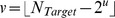
(18)

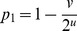
(19)

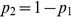
(20)


where 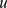 is the number of divisions in the first division cycle, 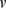 is the difference between the target number of neurons (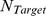) and the number of neurons resulting from the first division cycle, and 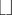 denotes the floor function for rounding to integers.

The intrinsic production constant 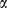 determines the number of cell divisions until differentiation into excitatory and inhibitory neurons can occur. The higher it is, the faster the 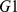 gene reaches the threshold of 0.99. 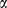 was adjusted manually in order for 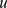 divisions to occur in the 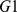 cycle.

### Development of Neuronal Morphology

Initially, neuronal cell bodies are assigned uniformly random positions in 3D unprepared space. In Cx3D, these cell bodies are modeled as physical spheres. The neuronal cell density was in agreement with values derived from experimental data, i.e. in the range of 40'000 to 86'900 per mm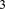 [119]–[121]. We found 250 neurons (200 excitatory and 50 inhibitory) to be appropriate for the available computer resources. For the establishment of neuronal connectivity, the somata were placed randomly in a cube with side length 160 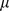
m. A smaller network of 150 neurons in a cube with side length 140 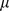
m was used for simulations where the second developmental phase was simulated, in order to decrease simulation time. 3 of these 150 neurons did not get inhibitory inputs after the initial outgrowth and were not included for the simulation of learning, such that the analyzed network consisted of 117 excitatory and 30 inhibitory neurons. Standard Cx3D parameters for the physical properties of the cells (e.g. mass or adherence) were used [16]. The somatic diameters were set to 8 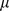
m. Variation of these parameters had only minimal effects on the simulation results.

Axonal and dendritic growth were encoded with the instruction language G-code [24]. We used the following mechanisms, which are executed by such G-code “modules” located in the growth cone, for axonal and dendritic growth, as well as synapse formation:

#### Axonal growth

The axon is initially extended from the cell body in a random direction, and is dependent on the concentration of an extracellular substance that is secreted by the somata of the neurons participating in the WTA network. The growth cones of excitatory and inhibitory neurons sense the substance secreted by inhibitory and excitatory neurons, respectively. As long as the concentration of this substance is higher than a given threshold 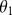, the axonal growth continues (see below). If the growth cone enters a zone where the concentration is below the threshold 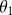, then the axon retracts until the concentration is above another threshold 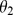 (with 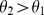), before resuming elongation. During elongation, the direction at each time step is a weighted sum of the direction from the previous time step, and of a random perturbation (i.e. 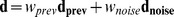). At each time step, the axon bifurcates with probability 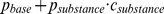, where 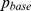 and 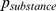 are constants and 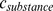 is the substance concentration at the current location of the growth cone. When the axon elongates or branches, its diameter is reduced by a factor 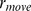 or 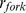, respectively. The outgrowth stops when the axonal diameter falls below 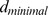.

#### Dendritic growth

Each soma produces three dendrites. As for the axon, the initial sprouting direction for dendrites is random. The subsequent elongation direction is also chosen as a weighted sum of the previous direction and a random perturbation. The major difference is that dendrites are not sensitive to extracellular cues (and do not retract). Branching and stopping mechanisms are implemented as in the axonal case. As a result, the overall dendritic morphology develops isotropically.

#### Synapse formation

In Cx3D, synapses are modeled as connections between excrescences on neurite elements of axons (boutons) and dendrites (spines). For simplification, spines represent potential postsynaptic densities in general, such that we do not model the absence of spines in most classes of inhibitory neurons [56]. During elongation of the neurite, an excrescence is instantiated in the middle of the discretized element. Whenever the excrescence of the tip of a growing axon or dendrite is close to another excrescence, it can check whether they are of complementary types. This local process of synapse formation is done by means of a module that is instantiated in the most distal neurite element, which corresponds to the growth cone's location. If this condition of complementarity is fulfilled and the excrescences are close enough (i.e. the distance 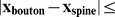 2 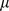
m, where 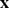 denotes the location where the excrescence is attached to the segment of the axon or dendrite), a synapse is formed. Synapse formation between neurites belonging to the same neuron (i.e. autapses) is prohibited, because their number has been reported to be relatively small [122], [123], and their electrophysiological significance is unclear.

In our Cx3D implementation, synapse formation also implies the establishment of a physical bond between the two excrescences on the axon and dendrite. This bond is approximated as a spring which reacts linearly to the force to which it is subject to. Therefore, connected neurite segments are kept close to each other, except when a certain repulsive force is exceeded. Once a bond is overstretched, it is released and the synapse destroyed. This ensures that synapses do not over-restrict the neuronal morphology.

The synaptic weights are assigned at synapse formation: Excitatory and inhibitory synapses are initialized with weights 0.001 and 0.01 respectively, in qualitative accordance with the estimate of [124]. The overall behavior of the simulations was not sensitive to these initial weights, because of the homeostatic adaptation processes.

### Electrophysiology

The computation of the electrical activity was implemented in Java, to allow a direct interfacing with Cx3D. All the synaptic weights in the Cx3D simulation are summed up, which yields a weight matrix. Based on this weight matrix, the input activity and the spontaneous activity, the firing rate of a neuron is computed according to Eq. 1. The numerical solution of the differential equations was computed using the explicit Euler integration method. The network's activity is computed with 3000 iterations and integration step 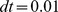. The maximal firing rate is set to 250 Hz.

#### Learning

In all the simulations of WTA learning, two different scenarios of input stimulation were conducted. In one case, the input neurons were activated in the form of a hill of activity. This hill was centered around an individual input population, and decayed with the distance from this center. The center population of the hill of activity was active with a rate of 1.4 Hz, the immediate neighboring populations at 0.5

1.4 Hz, the second at 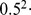
1.4 Hz, and the third at 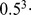
1.4 Hz. If the distance of the input population to the center population was bigger than 3, the activity of the input population was chosen to be a random number between 0 and 0.06. The hill of activity in the input layer moved in a periodic fashion (i.e. the peak restarts in the first input population of the input layer after having reached the last). This first scenario represents retinal waves, or orientation stimuli between 0 and 180 degrees. In the second case, discrete input patterns (Fig. 7A) are presented to the network. The stimulated input populations are active at a rate of 2.1 Hz, while the unstimulated ones are (as in the first scenario) active with a rate drawn uniformly from the interval 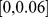 Hz. In both scenarios, the target average activity for excitatory neurons (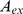) was identical for all excitatory neurons, and chosen between 0.14 Hz and 0.68 Hz. The inhibitory target activity was set to 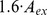. For the simulations shown in Fig. 8, target average activities of excitatory neurons were 0.4, 0.55 and 0.68 for the gains 1.3, 1.5 and 1.8, respectively. During learning, the input populations were active or not active in an alternating fashion. In the non-active case, only spontaneous and random activity generated electrical activity. This non-active input mode was introduced to demonstrate that correlated, instructive input can be intermittent in time. The neurons in the network have a random spontaneous activity that is drawn uniformly from the interval 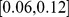 Hz.

Usually, the simulations took around 1 day to develop networks that exhibit WTA behaviour. The main computational bottleneck is the computation of the average activity, which relies on a large number of samples of neuronal activity. During an entire simulation, electrical activity in response to the input is computed around 1'000'000 times, and in the order of 100'000 learning steps (synaptic scaling and BCM learning) are performed.

The average activities used for the BCM learning (Eq. 3) are computed as the arithmetic mean of the neuronal activities from the last N inputs. N is set to 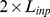, with 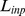 being the number of input populations. The factor 2 comes from the fact that the inputs are alternating between two different modi: active and non-active input neurons (as described before). In order for the WTA neurons to keep track of their activities, N has to be long enough to allow averaging the activities evoked by all possible inputs. Analogously, for the learning of the clustering based on discrete input patterns (second scenario), N was set to 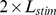, with 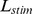 being the number of different input stimuli presented to the network. The time constant of the average activity is long compared to the time constant of the instantaneous firing rate, and assumed to be in the range of several hours to days. Based on this assumption, we assessed the real time of learning, and so some figures in this work indicate the estimated real-time of the learning process.

As a standard, we chose 20 input populations (or neurons) in the case of learning continuous input patterns, and the input strengths were initialized randomly and uniformly distributed between 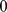 and 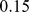. In the case of discrete input stimuli, we used 3 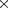 3 input populations. The initial connection strengths (from each of these input populations to each of the neurons in the grown network) were distributed in the range of [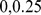]. We found little change of behavior when varying these numbers. During unsupervised learning, retinal-wave like activity or discrete input patterns are presented to the network in a periodic fashion. After every period, the new values of average activities are obtained and learning is simulated. The same synaptic learning time constants are used for input, excitatory and inhibitory lateral synapses. In the case of retinal waves, learning is simulated only after the hill of activity has passed every input population. In the case of learning discrete input patterns, learning is simulated each time a pattern is presented. The time duration for synaptic scaling after a retinal wave was chosen equal to the duration of learning one pass of all input patterns. Because of the 8 input patterns, 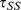 was set 8 times larger in the scenario of discrete patterns (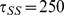 for retinal waves and 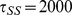 for discrete input patterns). The same time constants for the BCM learning rule were chosen, but exploratory simulations showed that this is not a necessary condition. In order to demonstrate that the WTA learning is not sensitive to changes in these time constants, simulations of WTA networks learning 4 stimuli were conducted with the same time constants as with 8 stimuli (S2A Figure). The weight change per learning step is constrained to maximally 3 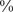 of the current value, in order to prevent large or destabilizing disruptions at the initial stages of development. The weight of an individual synapse between two neurons is approximated by dividing the total connection weight by the number of synapses involved in this particular connection. This absolute synaptic weight is constrained to a maximal value of 0.1 [125]. Overall, variation of the learning parameters do not have a strong effect on our results.

### Network Analysis and Visualization

Analyses of the simulated networks were performed with MATLAB (Mathworks Inc.). In order to assess WTA functionality, electrical activity was computed in the same way as in the Java implementation of the Cx3Dp simulation, namely using the rate-based model (Eq. 1) and the explicit Euler method. The integration step was decreased to 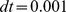 for minimizing integration errors.

The ordering of neurons for visualization, such as for Fig. 6A, was done using the genetic algorithm “ga.m” from the Global Optimization Toolbox of MATLAB. The energy to be minimized was defined as the sum of weighted topological distances between neurons, i.e. 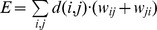, where 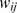 are the summed synaptic weights from neuron j to neuron i. The topological distances 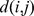 are inferred from a discrete 1-dimensional position vector of the neurons, which is initialized randomly and optimized. The ordering for the matrix visualization is then given by the locations of the neurons in this vector (i.e. neighbors in this vector are also neighbors in the matrix ordering). Note that the topological position is unrelated to the physical position of the neurons, and is only used for the optimization process. The visualization of the clustering was done with CytoScape [126], an open-source framework that is downloadable from http://www.cytoscape.org/. We used the “dynnetwork” plugin implemented by Sabina Pfister, which clusters weighted networks based on the Kamada-Kawai algorithm [127].

### Neurite Outgrowth Parameters

The neurite outgrowth has several parameters, which depend on the neuronal type (excitatory/inhibitory) and also on the neurite type (axon or dendrite). Table 1 lists all these parameters. The 2 substances which are secreted by the cell bodies and used by the axons as guidance cues both have a diffusion coefficient of 50 and a degradation constant of 5.

## Supporting Information

Figure S1 **Histograms of resulting numbers of neurons after simulation of the GRN.** The intrinsic instructions of the precursor cell in an unprepared environment lead to multiple neurons of two types (excitatory and inhibitory, other types like for example glia cells could facultatively be added). We conducted 100 trials of a GRN, that was set to give rise to 100 neurons, of which 80 are excitatory and 20 inhibitory. These results demonstrate that the (probabilistic) GRN produces approximately the desired number and proportion of neurons.
(TIFF)

Figure S2 **Visualization of network connectivity in weight space, after learning 4 input patterns.** (**A**) The locations of the neurons are determined using a clustering algorithm, such that strongly connected neurons are close to each other. Different colors indicate different preferred patterns of the neurons. The preferred pattern of a neuron was assessed by determining the pattern that evokes the largest electrical response. Inhibitory neurons are colored yellow and rectangular-shaped. The same network as in Fig. 7B and 7C is simulated, but after learning 4 input stimuli (horizontal, vertical and both diagonally oriented bars) instead of 8. The 4 clusters defined by the spatially proximal assemblies of neurons are visible. Importantly, the same parameters (time constants of synaptic scaling and BCM learning) were used, demonstrating the robustness of the learning scheme. (**B**) Network connectivity after using the same BCM learning rule both for excitatory and inhibitory neurons. As in (A), the locations of the neurons are determined using a clustering algorithm, such that strongly connected neurons are close to each other. Different colors indicate different selectivities of the neurons. In contrast to the simulations where synapses onto inhibitory neurons were following the synaptic scaling rule, here we used exactly the same learning dynamics for both types of neurons. This influences the clustering, such that also the inhibitory neurons become selective for the learning input stimuli.
(TIFF)

Figure S3
**WTA competition between two populations after correlation-based BCM learning of excitatory and inhibitory neurons.** WTA populations compete for representation of a mixture of 2 concurrent stimuli. In contrast to the simulations for Fig. 8, synapses onto excitatory as well as inhibitory neurons followed the BCM rule during learning. Also in this case, the populations with different preferred stimuli compete and mutually suppress each other. The blue crosses indicate samples of the relative activity of a WTA population selective for one of the two concurrent stimuli. The continuous blue line is the interpolation of these samples. The dashed blue line indicates the relative activity of the competing population. The green line is the angle bisector given by 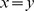. The horizontal and vertical axes show the relative contribution of two concurrent stimuli (two orthogonal orientations) and the corresponding populations (see legend of Fig. 8 for a detailed description). If there was no competition between the populations, simulation samples would lie on the green line, because then the network simply mirrors its input.(TIFF)

Video S1 **Neurite outgrowth in the 2-dimensional plane, with concentration-dependent axonal retraction.** Excitatory and inhibitory neurons (colored red and purple, respectively) are initially randomly positioned on a 2-dimensional unprepared environment. The somata of both types secrete different substances, which are sensed by the growth cones at the tip of the axons. Whenever the sensed concentration falls below a predefined threshold, axons retract until they reach a high enough concentration (this retraction is indicated in green). This behavior is iteratively instantiated, allowing the network to project more efficiently, because axons do not grow into regions where no potential targets are located.
(WMV)

Video S2 **Clustering of functional connectivity in a WTA network.** The presence of functional connections among excitatory and inhibitory neurons (red and blue respectively) are indicated with arrows. For clearer visualization, the strength is not shown. A clustering algorithm was applied to move the nodes such that strong connections are more probable to be close to each other. Therefore, the video does not show any physical movement, but only the arrangements performed by the clustering algorithm in weight space. 4 input stimuli referring to horizontal, vertical and both diagonal orientations are presented to the network. In the first part of the video (until 0:07 min), all neurons do synaptic scaling. Subsequently, synapses onto excitatory neurons become subject to the BCM learning rule, which has impact on the clustering of the functional connectivity: 4 clusters emerge for excitatory neurons, in contrast to the inhibitory neurons. This discrepancy is because of the different learning rule simulated after the first part, which is BCM learning for synapses onto excitatory and synaptic scaling for synapses onto inhibitory postsynaptic neurons.
(MOV)
