# Supplementary figures and images for: Developmental Self-Construction and -Configuration of Functional Neocortical Neuronal Networks
Source: PLoS Comput Biol. 2014 Dec 4;10(12):e1003994. doi: 10.1371/journal.pcbi.1003994 (PMC4256067; doi:10.1371/journal.pcbi.1003994)

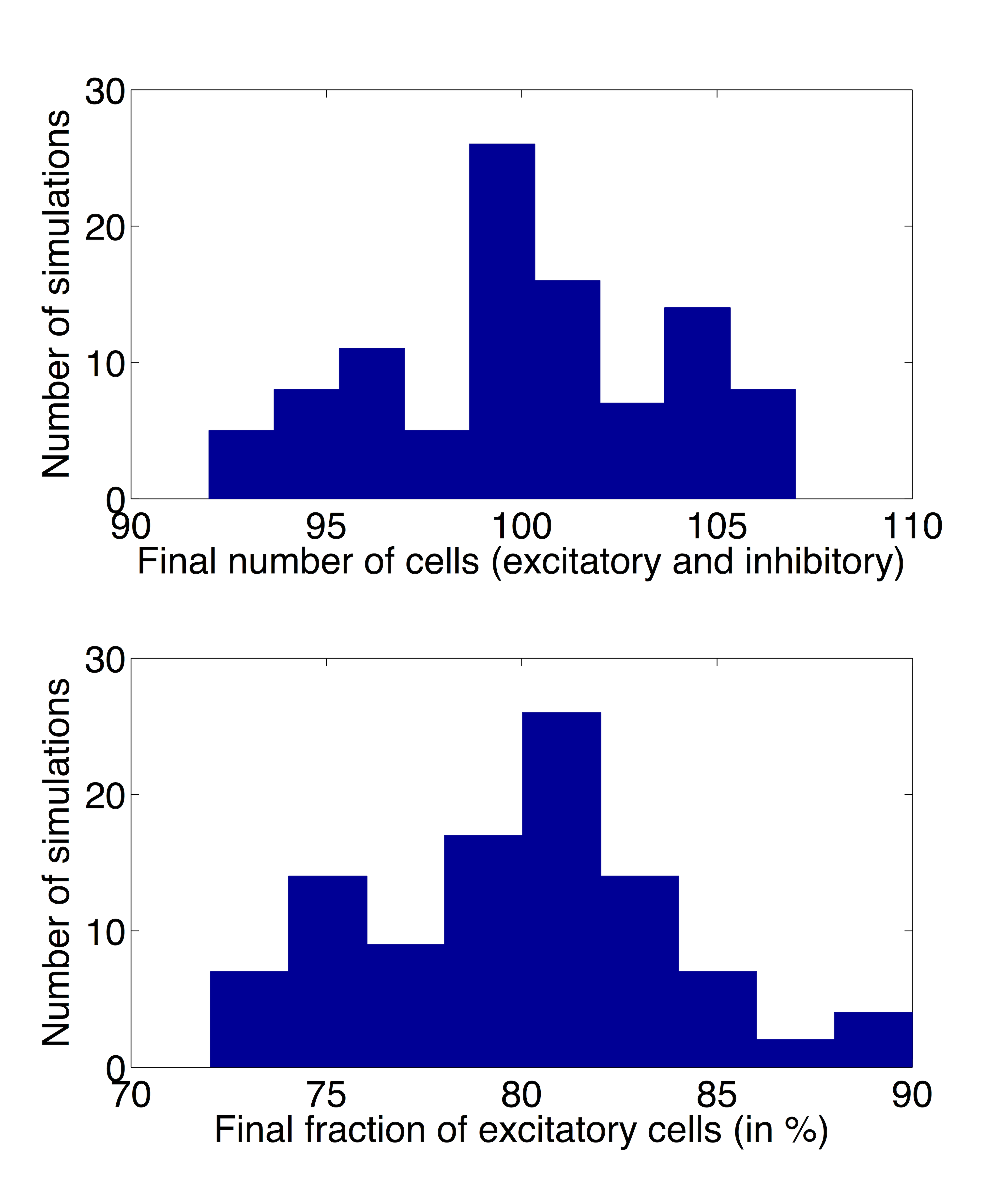

Supplement: Figure S1 — Histograms of resulting numbers of neurons after simulation of the GRN. The intrinsic instructions of the precursor cell in an unprepared environment lead to multiple neurons of two types (excitatory and inhibitory, other types like for example glia cells could facultatively be added). We conducted 100 trials of a GRN, that was set to give rise to 100 neurons, of which 80 are excitatory and 20 inhibitory. These results demonstrate that the (probabilistic) GRN produces approximately the desired number and proportion of neurons. (TIFF) [file pcbi.1003994.s001.tiff]

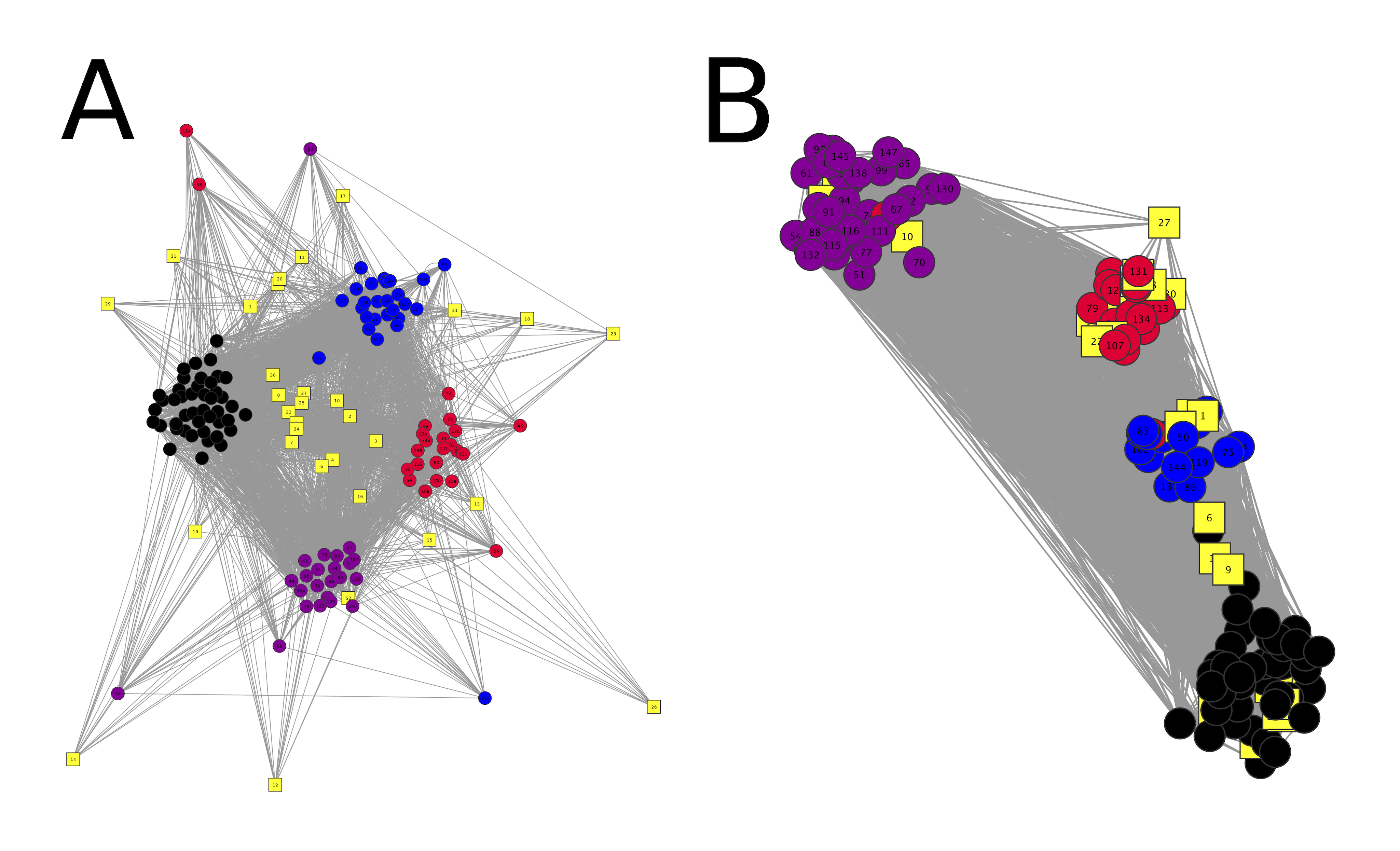

Supplement: Figure S2 — Visualization of network connectivity in weight space, after learning 4 input patterns. (A) The locations of the neurons are determined using a clustering algorithm, such that strongly connected neurons are close to each other. Different colors indicate different preferred patterns of the neurons. The preferred pattern of a neuron was assessed by determining the pattern that evokes the largest electrical response. Inhibitory neurons are colored yellow and rectangular-shaped. The same network as in Fig. 7B and 7C is simulated, but after learning 4 input stimuli (horizontal, vertical and both diagonally oriented bars) instead of 8. The 4 clusters defined by the spatially proximal assemblies of neurons are visible. Importantly, the same parameters (time constants of synaptic scaling and BCM learning) were used, demonstrating the robustness of the learning scheme. (B) Network connectivity after using the same BCM learning rule both for excitatory and inhibitory neurons. As in (A), the locations of the neurons are determined using a clustering algorithm, such that strongly connected neurons are close to each other. Different colors indicate different selectivities of the neurons. In contrast to the simulations where synapses onto inhibitory neurons were following the synaptic scaling rule, here we used exactly the same learning dynamics for both types of neurons. This influences the clustering, such that also the inhibitory neurons become selective for the learning input stimuli. (TIFF) [file pcbi.1003994.s002.tiff]

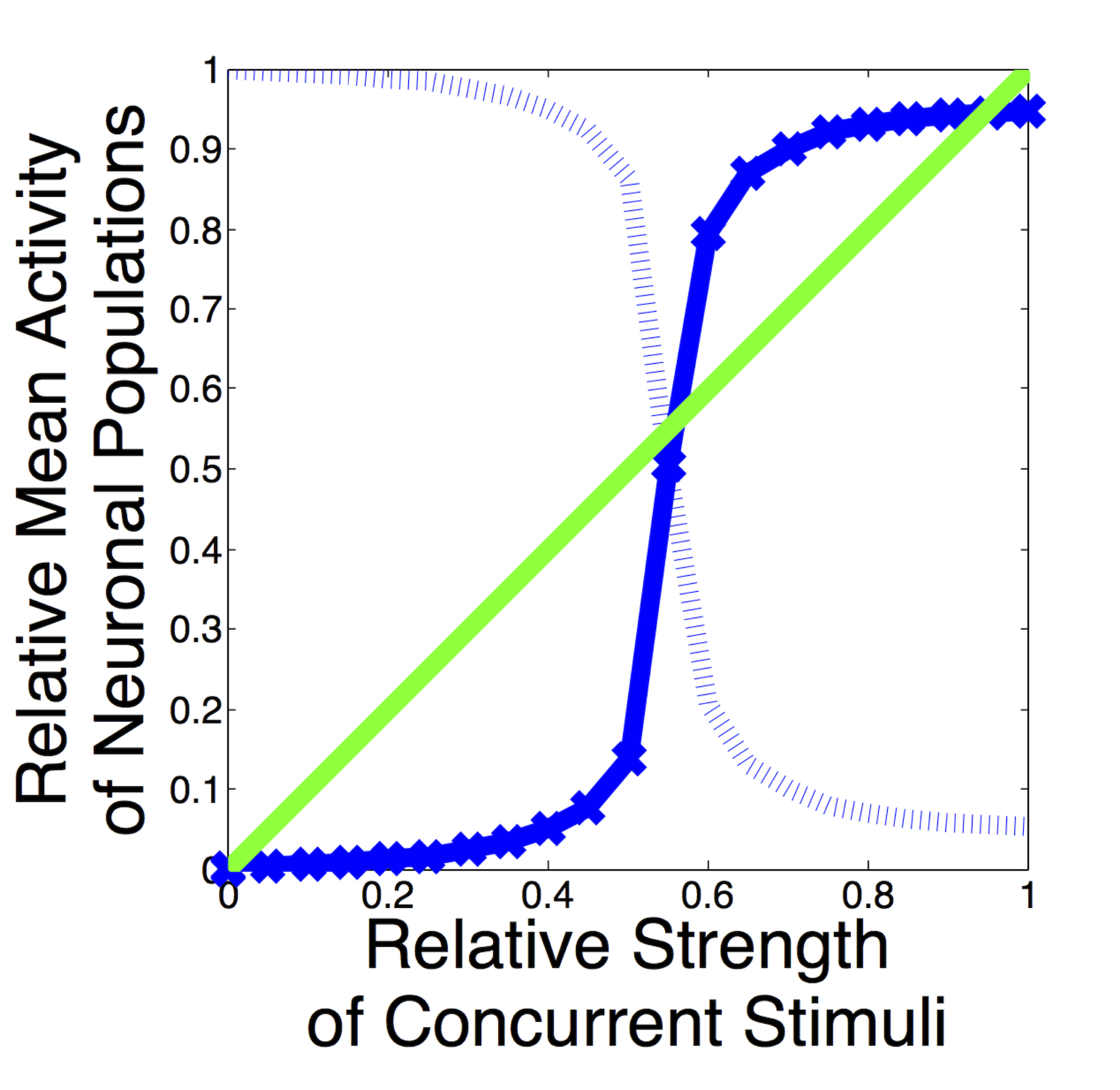

Supplement: Figure S3 — WTA competition between two populations after correlation-based BCM learning of excitatory and inhibitory neurons. WTA populations compete for representation of a mixture of 2 concurrent stimuli. In contrast to the simulations for Fig. 8, synapses onto excitatory as well as inhibitory neurons followed the BCM rule during learning. Also in this case, the populations with different preferred stimuli compete and mutually suppress each other. The blue crosses indicate samples of the relative activity of a WTA population selective for one of the two concurrent stimuli. The continuous blue line is the interpolation of these samples. The dashed blue line indicates the relative activity of the competing population. The green line is the angle bisector given by . The horizontal and vertical axes show the relative contribution of two concurrent stimuli (two orthogonal orientations) and the corresponding populations (see legend of Fig. 8 for a detailed description). If there was no competition between the populations, simulation samples would lie on the green line, because then the network simply mirrors its input. (TIFF) [file pcbi.1003994.s003.tiff]
